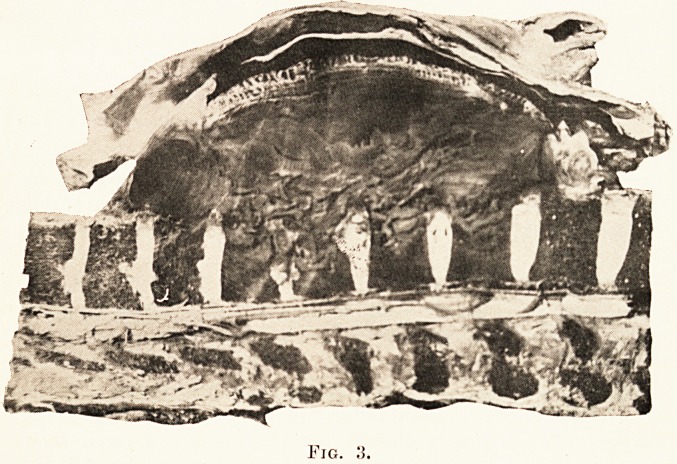# Meetings of Societies

**Published:** 1929

**Authors:** 


### Meetings of Societies.

### Bristol Medico-Chirurgical Society.

The Annual Meeting was held in the Physiological Theatre at the University on 9th October, 1929, the President and sixty-four members and guests being present. Dr. H. L. Ormerod resigned the chair to his successor, Mr. C. Ferrier Walters, who delivered his Presidential Address on **"The Duty of Medicine to the Race."**

The Secretary's report and the Treasurer's balance sheet were presented and accepted. Dr. A. L. Flemming read the report of the Editor of the *Journal.* The following officers were elected :—

Committee.

*President*—Mr. C. Ferrier Walters, F.R.C.S.

*President-Elect*—Mr. D. C. Rayner, F.R.C.S.

*Ex-officio.*

*Hon. Secretary*—Mr. W. A. Jackman.

*Hon. Medical Librarian*—Dr. W. A. Smith.

*Hon. Treasurer*—Mr. A. E. Iles.

*Editor of the Journal*—Dr. J. A. Nixon.

*Elected.*

Dr. H. L. Ormerod.

Dr. A. L. Flemming.

Dr. W. Kenneth Wills.

Mr. Duncan Wood.

Dr. R. S. S. Statham.

Mr. H. Chitty.

Mr. C. H. Terry.

Mr. Hey Groves.

Mr. H. G. Kyle.

*University Library Sub-Committee.*

Dr. W. A. Smith. Dr. G. Parker. Mr. A. R. Short.

### BRISTOL MEDICO-CHIRURGICAL SOCIETY.

### INCOME AND EXPENDITURE ACCOUNT for the year ended 30th September, 1929.

### INCOME.

| | £ | s. | d. | £ | s. | d. |
|---|---|---|---|---|---|---|
| Members' Subscriptions .. | 321 | 4 | 0 | | | |
| Do., outstanding for 1928–1929 .. .. .. .. .. | 12 | 12 | 0 | | | |
| | | | | 333 | 16 | 0 |
| Interest on Bank Deposits .. .. | | | | 11 | 7 | 9 |
| Interest on £400 4 per cent. Consolidated Stock .. .. .. .. .. | | | | 12 | 16 | 0 |
| | | | | £357 | 19 | 9 |

### EXPENDITURE.

| | £ | s. | d. | £ | s. | d. | £ | s. | d. |
|---|---|---|---|---|---|---|---|---|---|
| Grant to *The Bristol Medico-Chirurgical Journal* .. .. .. | | | | | | | 247 | 14 | 0 |
| Librarian—Honorarium .. .. .. | | | | | | | 7 | 10 | 0 |
| Miscellaneous Expenses— | | | | | | | | | |
| Fortts—Refreshments .. | | | | 5 | 5 | 0 | | | |
| Postages and Sundries | | | | 8 | 10 | 0 | | | |
| Printing and Stationery | | | | 7 | 5 | 0 | | | |
| Audit Fee .. .. .. | | | | 2 | 2 | 0 | | | |
| Lantern and Cinematograph at Meetings .. | | | | 3 | 13 | 6 | | | |
| X-ray Viewing Screen (½ share) .. .. .. | | | | 9 | 0 | 0 | | | |
| Lewis's Medical and Scientific Library— | | | | | | | | | |
| Subscription | 4 | 4 | 0 | | | | | | |
| Postages .. | 0 | 16 | 4 | | | | | | |
| | | | | 5 | 0 | 4 | | | |
| Wreath for Mr. Eycott .. | | | | 2 | 2 | 0 | | | |
| | | | | | | | 42 | 17 | 10 |
| Balance, being excess of Income over Expenditure carried to next Account | | | | | | | 59 | 17 | 11 |
| | | | | | | | £357 | 19 | 9 |

### BALANCE SHEET as at 30th September, 1929.

### CAPITAL AND LIABILITIES.

| | £ | s. | d. | £ | s. | d. |
|---|---|---|---|---|---|---|
| Sundry Creditors .. .. | | | | 84 | 14 | 0 |
| Capital as at 30th Sept., 1928 .. .. .. .. .. | 556 | 12 | 9 | | | |
| *Add* Arrears of Subscriptions paid up .. .. | 4 | 4 | 0 | | | |
| | 560 | 16 | 9 | | | |
| Balance of Income and Expenditure Account as above .. .. .. .. .. | 59 | 17 | 11 | | | |
| | | | | 620 | 14 | 8 |
| | | | | £705 | 8 | 8 |

### ASSETS.

| | £ | s. | d. | £ | s. | d. |
|---|---|---|---|---|---|---|
| Cash at Bank— | | | | | | |
| Current Account .. .. | 163 | 18 | 2 | | | |
| Deposit Account .. .. | 174 | 17 | 6 | | | |
| | | | | 338 | 15 | 8 |
| £400 4 per cent. Consolidated Stock at cost .. .. .. .. .. .. .. .. | | | | 354 | 1 | 0 |
| Members' Subscriptions for 1928–1929 outstanding .. .. .. .. .. .. | | | | 12 | 12 | 0 |
| | | | | £705 | 8 | 8 |

Audited and found correct,

H. E. KEELER, F.C.A.

16–18 Clare Street, Bristol,

*7th October*, 1929.

### British Medical Association.

(BATH AND BRISTOL BRANCH.)

At a clinical meeting held in the Medical Library of the University of Bristol on Wednesday, 30th October, a large number of cases was shown. The following is a brief account of these, also of radiograms and pathological specimens demonstrated at the same meeting :—

Mr. Wilfred Adams showed the following cases :—

1. **Carcinoma of the Stomach.** Three cases treated by partial gastrectomy. The first was that of a man aged 59,

A.= Growth.

B.= Simple ulcer which is amenable to this type of operation.

Shaded area is the small portion left after excision and forms the new stomach.

Dotted line " X " represents line of division of stomach sewn up to constitute a new lesser curve.

" Y " represents end of divided stomach making new pylorus, and was joined to duodenal stump.

who gave a history of seven years' vague abdominal pain with severe hæmatemesis four months before he was first seen. Hemorrhage continued to recur for several months. On palpation there was swelling and resistance in the upper abdomen, and an opaque meal showed a filling defect in the pyloric end of the stomach with delay in emptying. After blood transfusion a partial gastrectomy was performed. Two months later he had regained strength, but was still liable to much pain at times.

The second patient, a man aged 63, had for three months had no appetite, and in that period had lost two stone. There had been neither nausea nor vomiting. A lump was visible under the upper left rectus. At operation a bun-sized nodular cancer of the pyloric half of the stomach was found and removed by partial gastrectomy. Recovery from the operation was satisfactory.

In the third case, that of a man aged 64, there was a history of three months' dyspepsia, with rapid loss of weight. A lump was easily felt, and the opaque meal showed a gross filling defect, with delay, at the pylorus. This growth also was removed by partial gastrectomy, and two months later the patient was doing well.

The technique employed was a modification of Schumacher's.

2. **Mediastinal Growth.** The patient, aged 26, had for two weeks slight pain and fullness in the right neck, with dyspnœa on exertion. The face and neck were puffy and congested, the veins were large, and there was a fullness palpable in the neck, but no tumour. On exertion there was obvious dyspnœa. The skiagram showed a large mass in the right upper chest. Treatment by deep X-rays had been instituted.

3. Three cases of **Ankle Injury** treated by open operation. The first patient, a woman aged 30, displayed an unreduced posterior dislocation of the ankle seven months after sustaining a Pott's fracture. At the operation the fibula was cut through, the foot brought forward, and the tibial fragments screwed. The anatomical result was good, and the foot was working well nearly two years later.

The second case, that of a heavy woman aged 36, was similar in character, and was treated according to the same plan, except that the tendo Achillis had to be divided. Three months later she could walk short distances only.

In the third case, that of a man aged 25, the injury was a Pott's fracture eight weeks old. Here also open operation

PLATE XXVII.

Fig. 1.

Fig. 2.

Fig. 3.

Fig. 4.

was practised, the functional result remaining satisfactory eighteen months later.

4. Three cases of **Fracture treated with Ivory Pegs,** in each instance with excellent anatomical and functional results. The mandible, the upper end of the right humerus, and the lower end of the right humerus were the bones concerned in these three cases.

5. **Disease of the Urinary Tract to demonstrate the value of Pyelography.** Of the four pyelograms shown, two were from cases of renal tuberculosis and indicated conditions were confirmed after nephrectomy, the functional results being satisfactory. In another, a case of post-partum pyelitis, the pyelogram showed the left kidney lying on part of the sacrum, whence it was successfully removed. In a fourth case pyelography proved that the right ureter was double and the left blocked by a stone.

DR. J. A. BIRRELL showed the following cases :—

1. A case of **Paget's Disease of Bone.** Mrs. M. M., aged 63, first seen in March, 1928, complained of shortness of breath with retrosternal oppression on exertion. She was well nourished and of good colour. Auscultation of an enlarged heart revealed an aortic systolic murmur and a second sound of poor quality. The feet were œdematous, the blood-vessels distinctly thickened. The blood-pressure was 150/80. As she turned away her gait was observed to be "waddling," and when questioned about this she confessed to pains in the shin for several years and headaches.

Examination of the skeleton revealed a head of circumference 24 inches and marked bossing over the upper forehead, so that she had to wear the largest available hats ; very thickened and rounded clavicles ; the ribs disposed as in advanced emphysema ; the tibiæ rounded so that the crests might not easily be defined, the shafts withal curved with a forward and outward convexity. (Plate XXVII., Figs. 1, 2, 3.)

X-ray photographs taken by Dr. G. B. Bush revealed bony changes typical of Paget's disease of bone. The Wassermann reaction was negative. With the progress of time the dyspnœa and unnatural gait have rendered walking more difficult, so that now she is wheeled about in a chair. Moreover, there appears to be a gradual vascular deterioration as gauged by a progressively rising blood-pressure, which to-day is 180/100. Within the last few months she has developed a copious glycosuria with a blood sugar of 2 per cent. on an ordinary diet.

There are two interesting features in the case. There is the fact that she complained of cardiac symptoms only, leaving those of the skeletal deformities in the background, so that the primary symptomatic diagnosis was that of atheroma. This is apparently often the case in the diagnostic history of Paget's disease. There is furthermore the hyperglycæmia ; diabetes insipidus is frequently associated with osteitis deformans, but the onset here of hyperglycæmia provokes the speculation as to the pituitary gland being accountable both for the abnormalities of sugar metabolism and of skeletal design.

2. A case of **Pancreatic Infantilism.** E. M., aged 13½, was admitted to the Children's Hospital in June, 1928. She had not progressed physically or mentally since the age of 5. She talked with the intelligence of a girl of 5, and with the exception of a partiality towards drinking water from flower vases and swallowing fluff picked from her blankets, she had been clean in her habits. She had been able to feed herself quite well, but had a great aversion to fats ; any fat in her diet provoked vomiting. She could hardly move a limb, lying listless and displaying no interest in her surroundings.

At first sight her appearance suggested progeria (Plate XXVII., Fig. 4) : a little weak and wizened woman, prematurely senile ; her skin dry, wrinkled and transparent ; her hands shrivelled and almost weblike ; her face shrunken, pallid and with a shy but precocious expression ; the hair greyish, listless and dry, lacking sheen, and eyes sunken in browless sockets ; the limbs generally gave the impression of bony stalks, lying amidst fatless and stringlike muscles, loosely enclosed in a sheath of parchment-like skin. There was a complete lack of sexual development. She weighed 1 st. 8 lb., and her height was 3 ft. 5 in. The Wassermann test was negative. There was no relevant family history.

The cardiovascular system appeared normal, with a blood-pressure of 90/65—there was no suggestion of atheroma, which is a characteristic of progeria, and the urea tests were normal.

The abdomen was extremely distended and drumlike in its tenseness ; while a very large colon as revealed by skiagraphy, and the passage of very bulky and offensive pale stools of the consistency and appearance of soft putty, were features casting suspicion on the pancreas or colon. A diagnosis of pancreatic infantilism was tentatively made.

The urine contained no sugar; the glucose tolerance and diastatic tests were satisfactory. The dried fæces contained a considerable excess of fat, with 66 per cent. of unsplit fat in the total fat content.

X-ray examination of the skeleton showed a great lack of density of the long bones, with no evidence of rickets or pituitary fossa abnormalities.

To test the truth of a lack of tolerance to fats, she was at first put on a diet of extra cream and milk; the stools at once became much more bulky and offensive, and there were vomiting attacks. The diet was now replaced by one containing no fresh milk or fat: she was fed on condensed milk, rusks, meat *ad lib.*, and half an ounce of meat juice daily, with some bread and vegetables.

As regards drugs, thyroid was at first tried, to exclude the possibility of an unrevealed deficiency of this gland: this brought about no improvement. With her fat-free diet a drachm of Benger's pancreatic extract was given three times in the day, and half an ounce of Armour's liver extract at mid-day and evening—this line of treatment has been continued for the last twelve months.

There has been a slow but consistent improvement, with the exception of a series of tetany-like convulsions during a week in January, 1929. She is now able to walk about and plays with other children. The stools are practically normal; the abdomen much less distended; the general trophic condition is much improved and her weight is 2 st. 3 lb. She has, however, grown but 2 in. in height, and sexually there has been no advance.

MR. E. R. CHAMBERS showed the following cases:—

1. A case of **Dermoid Tumour on the Sclero-corneal margin** of a baby. It was whitish in colour, raised, its surface epidermoid and dry, sometimes covered with fine down or even hair. Histologically these growths possess the composition of external skin, they consist of a stroma of connective tissue covered with epidermis and contain hair follicles and glands. They are congenital and tend to increase in size. They can be shaved off the surface of the eye.

2. A case of **Rodent Ulcer of the Upper Lid** in a man aged 51. This was first noticed a year ago, but had probably existed much longer. It was treated with three doses of deep X-ray therapy. This completely healed it, but it subsequently broke down over an area larger than it had to start with. Many doses of surface radium were then given. This healed it, but it has again broken down, and it is extending towards the inner canthus. Mr. Chambers suggested (1) that the lid should be removed and the orbit eviscerated, or (2) that radium needles should be used.

3. A case of **Tubercle of the Lachrymal Sac** occurring in a girl aged 24. Pathological investigation confirmed the diagnosis. The sac was opened and scraped and packed with "bipp." Tuberculin injections were given over a long period. Tubercle of the lachrymal sac is a very rare condition, and is most difficult to treat. It is probable that the tuberculin played a considerable part in the cure, and that it should always be given a trial in this type of case.

MR. H. CHITTY showed the following cases :—

1. A woman who was first seen in August last suffering from **Carcinoma of the Left Breast.** Malignant glands were present in both axillæ, but no tumour could be felt in the right mamma. The left breast was removed, and both axillæ cleared. When the patient was seen early in October there were recurrent nodules of growth in the operation scar, and a very large mass could be felt in the right breast, which was red and œdematous. The case is being treated by deep X-ray therapy.

2. A child aged 6 with **Cleft Hands.** Both middle fingers are absent, and skiagrams show on the right side fusion of the second and third metacarpals, the cleft passing between the third and fourth metacarpals, while on the left side it passes between the second and third. A maternal uncle has a similar condition of one hand but without suppression of any digit, the cleft passing between the third and fourth metacarpals. The condition as a rule shows a marked hereditary tendency.

DR. R. C. CLARKE showed a case of **Mammary Hypertrophy** in a woman aged 64, a nullipara. This condition, which was bilateral, had not been noticed till five years ago.

MR. J. ANGELL JAMES showed a case of **Carcinoma of the Larynx treated with Radium.** The patient was first seen in November, 1928, complaining of hoarseness and loss of voice for eighteen months. On examination a fixed whitish nodular mass was seen, in the position of the right vocal cord, extending anteriorly to the anterior commissure. posteriorly to the arytenoid, laterally to the ventricular band. The pathological report on the specimen removed by direct laryngoscopy showed it to be a squamous carcinoma. At an operation, after fenestration of the right thyroid cartilage, nine milligramme radium needles were inserted. The radium was removed one week later. His voice is now as strong and clear as before the symptoms appeared. The right vocal cord is thicker than the left, and moves freely, but not as well as the left.

DR. S. HARDY KINGSTON showed :—

1. A series of cases and photographs illustrating **Occupational Dermatitis and its Sequelae.** These included examples of arsenical dermatitis ; epithelioma in an anthracene worker ; epithelioma of the wrist in a taxi-driver, a similar lesion in a tar worker, also epithelioma of the lip in a tar worker, and a " tar wart " of the forearm ; an epithelioma of the face in a farmer, and one on the hand of a farmer ; epithelioma caused by Rangoon oil on the ear and nose ; epithelioma of the scrotum in a sweep ; epithelioma on the thumb of a baker ; epithelioma of the tongue in a cobbler ; multiple rodent ulcer in a X-ray worker, and also X-ray dermatitis and tinea in a X-ray worker ; " barley itch " (trunk) ; mayweed dermatitis ; Japanese oak dermatitis ; bomb dermatitis ; oil acne ; benzene eruption on the hands of a cleaner of golf balls ; dystrophy of nails in an orange cutter ; and " chrome hole " in a colour worker.

2. A case of **Ichthyosis**, a congenital hypertrophy of the horny layers of the skin, in which the skin is dry, scaly and looks dirty. This disease appears in the first year, and increases in severity from the fifth to the fifteenth, then persists throughout life. The roughness of the skin causes adhesion of particles of dirt and dust, so that parents complain it is impossible to keep the parts clean. Thyroid will sometimes improve the condition. Frequent bathing is useful to remove the scales. In mild cases equal parts of glycerine and water applied once daily will keep down the scaliness and impart smoothness to the skin. In the more severe forms equal parts of olive oil and lanoline rubbed in after a daily bath are helpful.

3. A case of the **Premycotic Stage of Mycosis Fungoides** in a man aged 55. Twelve years ago a patch of so-called " eczema " first appeared on the inside of the right thigh. The irritation was severe at all times. Since then this area has enlarged to quite twice its original size. In addition many other lesions have appeared on the chest, arms and legs. The plaques were red, dry, scaling, infiltrated and irregular in shape and size. Islands of healthy skin appeared in between these lesions. The spleen, hair and nails were not affected. The disease proved rebellious to all forms of local treatment except electric light baths, and at a later date regression of symptoms followed the application of X-rays. But the condition has now relapsed. A blood count was normal. A section made shows infiltration of cells in the corium with signs of growing down and thickening of the interpapillary layer. (This patient was shown at the Royal Society of Medicine two years ago.)

4. A case of **Dermatitis Herpetiformis.** This is a rare disease with four cardinal symptoms : (1) The eruption is polymorphic ; (2) it is attended with itching and sometimes with pain ; (3) it is recurrent ; (4) the patient's health remains good.

The eruption consists of groups of vesicles arranged in erythematous patches usually well defined of discoid shape or with a gyrate outline from the coalescence of several neighbouring lesions. The size of the vesicles varies much more than in herpes, and bullæ as large as a pea or nut are common. Eosinophilia may reach 20 to 90 per cent. of the cell elements of the fluid contents of the vesicle.

No region of the skin is exempt, the forearm perhaps more than other parts. On the subsidence of the eruption pigmented stains are left. There may be severe febrile phenomena, sometimes diarrhœa, but no general wasting as in chronic pemphigus. An attack may last for weeks or months. In some cases the eruption recurs throughout life. It is a most distressing disease, and it renders the patient unfit for long periods from pursuing his avocation, but it is not dangerous to life.

The treatment recommended consists of rest in bed, except in mild cases with a light occupation ; arsenic, quinine and antipyrin which are sometimes helpful ; a strict vegetarian diet with limitation of the quantity of fluid ; while autogenous vaccines, sour milk, high rectal douches all have their advocates. Dusting powders of talc, starch and zinc are useful when the blister formation is excessive.

5. A case of **Lupus Erythematosus,** of which there are two main types : (*a*) Circumscribed and chronic, constituting about

80 per cent. of the cases in this country; (*b*) a disseminated type, more or less acute.

This case is of the first type, which usually attacks the face where the skin is stretched and capillaries defective, as on the malar prominence, the bridge of the nose, and the ears. Note the rose-red, irregular shaped patches on the cheeks, replaced in some areas by thin whitish scars indicating retrogressive changes of an atrophic character.

The eruption has progressed slowly and relentlessly in spite of salicin and quinine internally, $CO_2$ externally, but in some other cases the administration of krysalgin has produced amelioration of the disease.

No local source of infection such as the tonsil was recognizable.

6. A case of **Lupus Vulgaris,** after treatment with acid nitrate of mercury.

There is profound disfigurement, ectropion, pale scarring of cheeks, the result of extensive ulceration, rather than of deep ulceration with bony destruction as in syphilis; also there is no involvement of the scalp as in lupus erythematosus.

7. A case of **Summer Prurigo.** Hutchinson's summer prurigo is a papulo-vesicular eruption affecting the face, neck and upper extremities. The history of its first appearance in early childhood, and the periodical recurrence in the summer, are usually sufficient to make a diagnosis from other pruriginous eruptions. Hutchinson advised arsenic in gradually increasing doses. Many take as much as six or seven minims of Fowler's solution thrice daily. Ichthyol internally has also been advocated. Any deviation from the general health should be attended to, and the lesions should be dressed with a soothing lotion, such as lead and tar.

8. A case of **Lichen Planus,** showing the characteristic eruption of lilac-coloured flat-topped papules varying in size from a pin's head to a millet seed or a little larger. White opalescent points or striæ forming a fine network are visible. This sign (first pointed out by Wickham) is pathognomonic. On the disappearance of the spots pigmentation remains. A prolonged course of arsenic appears to increase the latter. The eruption is rarely seen on the face, scalp or nails, but the mucous membrane of the mouth is affected in at least one half of the cases. The favourite site in the mouth is the inner aspect of the cheeks opposite the teeth, where the lesions are white porcelain-like patches of irregular shape or a network of z fine striæ. Itching is almost always present, and is worse at the beginning of an attack. The acute cases tend to clear up in a month or two. Occasionally the disease persists for years.

Treatment consists of freedom from worry and anxiety. Warm sedative baths are comforting ; sodium bicarbonate, a teaspoonful to each gallon of water, should be used. Locally X-rays in weak doses produce the best results. Salicin, arsenic and mercury are all of service. Menthol, camphor and phenol are also useful when applied locally.

9. A case of **Acne Rosacea.**

Professor J. A. NIXON showed the following cases :—

1. A young woman aged 32 who had recovered from **Ulcerative Colitis.** When seen on 5th November, 1928, she had been passing blood and mucous per rectum for five or six months, and had pain on the left side of the abdomen. Sigmoidoscopy showed the presence of ulcerative colitis. After three weeks of colon lavage she was still very ill and no improvement was noted. On 27th November transverse colostomy was performed by Mr. Short, and after colon washes with a solution of potassium permanganate she gradually improved. By April, 1929, the colostomy wound had closed, and on 22nd May she was discharged well.

2. A case of **Ulcerative Colitis** in a young woman aged 24, admitted on 13th July, 1929, complaining of pain in the abdomen for a week. She had had " piles " for one month. *Per rectum* round nodules were palpable in the mucosa or possibly outside the gut in the hollow of the sacrum. On 15th July sigmoidoscopy by Mr. Chitty showed the whole of the mucous membrane to be red and inflamed, with small polypoid growths of mucosa. Within reach of the finger were round nodules which were not fixed ; they appeared to be under the mucosa. Under treatment by colon washes the stools did not improve and still contained bright blood. On 9th August Mr. Chitty made a transverse colostomy, followed by colon washes through the colostomy with potassium permanganate 1/5000. A sigmoidoscopy by Mr. Chitty on 19th September showed the mucous membrane still inflamed and bleeding when the sigmoidoscope was passed. By 30th September the colon washes were clean.

3. A case of **Diabetes Mellitus** in a man aged 57, complicated by a left pyonephrosis which was drained on 21st July, 1929.

This was followed by development of an abscess in the right arm. On 29th August he was discharged from the Bristol Royal Infirmary, stabilized on insulin m 10 b.d., double strength. On 19th September he was re-admitted, with glycosuria and a gluteal abscess, which was drained. On 22nd October he was discharged stabilized on insulin m 15, double strength, b.d.

4. A case of **Crossed paralysis** in a man aged 45. On admission on 2nd October he complained of stiffness of the left side of the face and pain. He had had headaches for the last three or four months, and had vomited precipitately at rare intervals without nausea. He occasionally stumbled and tripped over one foot. There was no history of injury. On examination he showed left-sided facial paralysis and right-sided hemi-paresis. Blood-pressure: systolic 102, diastolic 70. His memory had not been good for the last six months; he had forgotten a very familiar verse of a song completely. Articulation was difficult. The spinal reflexes were more brisk on the right side than the left. The right side was weak. There were no objective sensory changes. He showed Bell's palsy on the left side, and his hearing was not so acute on the left as on the right side, but he complained of no deafness or tinnitus. His tongue deviated to the right. The fundi were not abnormal. The Wassermann test was negative. Cerebro-spinal fluid: cells 2 per cmm., globulin test negative, total protein 0·025 per cent., glucose 0·075 per cent. No micro-organisms in smears or cultures.

DR. PERCY PHILLIPS showed the following cases :—

1. A case of **Hereditary Deforming Chondrodysplasia.**

*Case.*—R.C., aged 25 years.

*Occupation.*—Infirmary attendant. This man came under medical observation when being examined for appointment. Weight, 9 st. 7 lb.; height, 5 ft. 9 in.

*History.*—First noticed "lumps" on legs at age 5. These have grown steadily since, but not during the last four years. No serious illnesses.

*Complaining of.*—No symptoms. Can play tennis and other games. Extremely sensitive about his deformities, and will not attend to be seen. Has carried out his duties satisfactorily for the past twelve months.

This disease, sometimes known as multiple congenital osteochondromata, is probably an hereditary disturbance of metabolism of cartilage and bone.

*Clinical features.*—It is three times as common in males as in females, begins in early life, and is characterized by the appearance of multiple growths in the bones. These growths are merely incidental and not the essence of the disease. The bones most commonly affected in order of frequency are the femur, tibia, humerus, fibula, radius, ulna, phalanges, ribs, scapula, and pelvic bones. Face and skull are rarely involved. The changes may be divided into two main groups : (*a*) growth retardation ; (*b*) proliferative changes.

SITES OF CHONDROMATA,

(*a*) *Growth retardation.*—This may affect form, length or thickness of bone. The bones may be normal in length but remain thin and delicate. Cyst formation may be visible in the X-ray. Under-development of the fibula may give rise to a condition of pes valgus, whilst that of the radius may cause deformity of the wrist with ulnar deviation.

(*b*) *Proliferative changes.*—These have been observed both distal and proximal to the epiphyseal line. Early disappearance of this line is a striking feature of the X-ray. In the epiphyses there is enlargement and distortion of the bone. Outgrowths, sometimes stalactite-like in form, may encroach upon epiphyses but originate in the diaphysis. In the shaft of the diaphysis appear a number of definite exostoses. These often appear as a result of injury. It appears as if the bone were specially sensitive to injury and reacts in this way. The disease ceases when skeletal development is complete.

*Pathology.*—The nature of this disease is a disturbance of bony metabolism occurring early in life, or perhaps *in utero.*

Honeij states : " In the stabilized stage of the disease calcium metabolism differs little from normal, whether the abnormal subject is maintained on a calcium-poor or a calcium-rich diet. In the progressive stage calcium metabolism is markedly different from normal in that calcium is lost from the body." Nests of cartilaginous cells may be left under the periosteum covering the ends of the shaft. These remain uncalcified, but at a later date they may develop into chondromata.

2. Two cases of **Syringomyelia.**

DR. A. T. TODD showed the following :—

1. A group of cases of **Malignant Disease treated with Lead Selenide.** This treatment is not in any way regarded as a cure for cancer. The cases accepted for treatment are all advanced and inoperable. Many of them have so extensive an invasion of tissues that recovery would leave them crippled, even if all the cancer was destroyed.

The object of this trial is to show that this colloid does act on cancer ; these few cases, chosen out of many, definitely show that there is an action. It is hoped to save life in cancer by the combination of this treatment with radium, X-rays and surgery in earlier cases.

But even in very advanced cases it is found that this colloid often causes marked prolongation of life, and that it often reduces the pain of cancer, even when morphine has no action.

(*a*) **Carcinoma of Ovary.** This case was shown to demonstrate the marked immediate improvement which lead selenide gives. It was a rapidly-growing carcinoma which spread over most of the peritoneum. There had been almost complete relief of symptoms, marked diminution of the masses of growth, considerable gain in weight and strength ; so much so that the patient has expressed a wish to be allowed to return to work.

(*b*) **Sarcoma of Ileum.** The patient was sent to Mr. Walters suffering from paralysis and anæsthesia of her right leg and thigh. She had a large hard mass which appeared to arise out of the pelvis on the right side. At operation this was found to be a large sarcoma, and removal was considered impossible. She was in fairly good general condition, had wasted little, and from the length of her history it was obvious that growth was slow.

Treatment by lead selenide started two years ago, and is still being given at long intervals.

Improvement was rapid. After the second injection there was a sudden return of sensation in the leg. This return was so sudden that it produced a nervous storm in the patient. The paralysis was more slow but steadily improved. The mass, which was about the size of a full-term pregnancy, gradually shrank, and by the end of a year was only just palpable. Now the mass cannot be felt.

The patient has gained much weight, has no pain, has completely regained sensation, and has so much gain in motility that she can walk several miles a day.

A point worthy of investigation in this case was the remarkable return of sensation.

(*c*) **Squamous Carcinoma of the Lip.** This man had a carcinoma of the lip removed at the General Hospital. A recurrence followed shortly afterwards, which was removed surgically. Recurrence took place again at a slightly longer interval. When he was sent to Dr. Todd the patient showed a small mass in the lip scar, two glands about the size of hazel nuts in the submaxillary region, and smaller glands down the neck. He had wasted, felt ill, and had a good deal of pain, especially on eating and smoking.

He was treated as an in-patient with $D_4S$, and showed rather rapid subjective improvement. More slowly the masses of growth first stopped increasing, and then began to disappear.

For over a year his condition has been as at present, no evidence of the former readily-visible masses is to be found. There is only some scarring of the sites of carcinoma. He has gained a good deal of weight and strength and has lost his dysphagia. Some feebleness and anorexia remain.

(*d*) **Squamous Carcinoma of the Tongue,** in a man aged over 70. When first seen about two years ago he had a very large cancerous ulcer of his tongue which had invaded the pillars of the fauces. The ulcer was the size of a penny. There were large glands under the jaw and in the neck, forming outstanding masses. He was ill, wasted and had considerable pain. His condition was so advanced that serious treatment was not thought profitable, but it was considered that treatment might diminish the pain.

He was treated throughout as an out-patient, going home an hour after injection of $D_4S$ each week. For about six months there was little change, but no extension of growth occurred. After that there was a slow diminution of both ulcer and glands, and before the end of a year all had disappeared.

Now there remains a painful scar which has deformed the tongue and pinches some of the sensory nerves. The present condition has been stationary for a year. He has gained much in weight and strength. His only troubles are some constipation and the pain around the ear. He received no treatment except injections of $D_4S$, *i.e.* colloidal lead selenide.

(*e*) **Carcinoma of Ovary.** Sent for treatment by Mr. Statham, and found to have a generalised carcinoma of peritoneum from a malignant ovary. The growth was so rapid that recurrence was found in the incision before healing had time to occur.

Rapid improvement followed lead selenide, and the masses quickly disappeared. A return of pain and reappearance of the masses was noted about nine months ago. On the whole there has been a marked improvement, enough for the patient to live at home and do some work for about a year.

Treatment has been much interfered with in this case by completely extraneous causes, and also because the patient has shown a dyspepsia with vomiting after injection. This is considered to be due to partial stenosis of several parts of the intestine due to fibrosis of regressed cancer. This type of cancer, *i.e.* that of the ovary, appears to be very easily attacked by lead ; in a number of such cases stenosis has followed and required short circuiting operations. These have all been very widespread cases, which would have survived only a short time without treatment. It is considered that less widely-spread cases will give good results with this treatment.

2. A case of **Lymphadenoma**. This condition is regarded as neoplastic by some and is included in the neoplastic diseases by Ewing. Here it is not so regarded, but is considered to be a disease closely related to the microbic granulomata. It has very close affinities to tuberculosis and to kala-azar. It is very little amenable to treatment, although a temporary betterment usually follows X-radiation. On the whole it is a rapidly fatal condition, and the mortality is greater than that of cancer.

This patient was sent by Dr. L. A. Moore. He complained of weakness, loss of appetite and fullness of the abdomen. On examination he was found to have a very large spleen, mediastinal glands and smaller gland masses about the neck, axillæ and groins.

He was given intensive arsenic treatment in the form of novarsenobenzol intravenously, dose 0·9 gramme, and by this time has been given 25 grammes. In addition he has had X-radiation applied to his liver four days after each injection. Of late he has been given a few doses of bismostab. He has done very well, the spleen has disappeared and the glands are much diminished.

The theory of this treatment is largely hypothetical. It is presumed that lymphadenoma is really a neoplastic disease, and that the site of "primary" growth is the liver. That this may be true in some cases may be the explanation why some of the cases do respond. So far, amongst about fifteen cases tried, only three have been at all a success.

DR. KENNETH WILLS showed the following cases :—

1. A case of **Lupus Erythematosus** treated by subcutaneous injection of sanocrysin (gold thiosulphate) in doses of 0·01—0·02 grammes weekly.

2. A case of **Pityriasis Rosea** in a girl aged 13. There was a typical herald patch, the exanthema appearing ten days later.

3. A case of **Lupus Vulgaris** of verrucous type on the nose, treated by pyotropin. There is now no active lesion present.

4. A case of **Rodent Tumour** of eleven years' duration, to show how slow growth is in the beginning, and how long a period may elapse before cases may come for treatment.

MR. E. WATSON-WILLIAMS showed a case of **Keratosis Pharyngis** with unusual distribution of masses, and said to be hereditary.

The patient, a young adult male, had noticed the appearance of white patches on his tonsils two months previously, when in good health. There was no pain, but a sense of discomfort, which has latterly become quite severe. The general health had been poor for a month, possibly on account of worrying about it. The patient states that his father had a similar condition " in white streaks " for many years, but it cleared up when he was about 45.

On examination are seen, on the left tonsil, a linear fringe of finger-like processes arranged along its posterior margin. These are about 2 to 7 mm. in length, about 2 mm. in thickness, and slightly club-shaped ; the smaller whitish, the larger brown towards the tip, or nearly black. On the right tonsil are three such processes, one above the other, and also one low patch. Usually there are a few low, slightly conical patches irregularly scattered about the surface of the tonsils, etc., with a tendency to symmetry as regards the two sides.

These projections are produced by an overgrowth of epithelial cells. They are firmly attached to the surface, and if removed, which requires firm scraping, a raw surface is left. The condition affects lymphoid tissue in the pharynx, most commonly the tonsils ; the lingual tonsil, naso-pharyngeal tonsil, and lymphoid patches on the back of the pharynx may all be affected, and a few cases have been recorded in which the larynx was involved. The symptoms are often inconspicuous, but a sense of harshness or discomfort in the throat is usual, not amounting to pain. The affection may be transient, spontaneously disappearing, or may last for months, or may recur. The general health is not affected, though keratosis is said to attack those in poor general health. Local removal of the masses is useless, and unless they are confined to the tonsils no local treatment should be attempted. If confined to the tonsils, and causing worry, the tonsils may be removed.

The ætiology is obscure. The condition must be differentiated from chronic lacunar tonsillitis—in this the cheesy masses are crypt exudate, and can be easily removed—from diphtheria, and from syphilis.

MR. DUNCAN WOOD showed a case of a boy aged 7 with **Baker's Cyst of Knee.** There was a history of two years' painless swelling of the right knee, with one month of swelling behind the right knee.

On examination the right knee was swollen and warm to the touch, fluid was present, there was free movement of the joint, and a fluctuating swelling in the popliteal space. The Wassermann reaction was negative. The X-ray showed irregularity of inferior articular surface of the femur. It was suggested that the cyst should be removed, as this would help in a diagnosis of the cause of the arthritis.

DR. F. J. A. MAYES showed a series of skiagrams from cases of **Congenital Deformity of the Hand; Disease of the Nasal Accessory Sinuses, Sphenoidal and Antral; Tuberculosis of the Lungs; Gastric Ulcer.**

DR. G. B. BUSH showed a series of skiagrams illustrating the methods of **Radiological Investigation of Diseases of the Gall-bladder.**

A complete examination includes :—

(i.) Careful radiography to show such stones as will cast a shadow, and with modern technique about 40 per cent. of all stones should thus be discovered.

(ii.) Special films to differentiate the shadows of gall-stones from the many other shadows that may be confused with them in this region.

(iii.) A search for indirect signs of gall-bladder pathology by means of an opaque meal, which may demonstrate reflex, pressure or traction effects on neighbouring viscera, such as stomach, duodenum or colon.

(iv.) The visualization of the gall-bladder and the study of its functions by cholecystography, a method which is now simple and free from unpleasant symptoms, and which will reveal the majority of stones which are not seen by straight radiography.

Skiagrams were shown demonstrating all these points.

In addition, three skiagrams were shown of a single case which, on investigation by an opaque meal, showed all of the following abnormalities :—

(1) A calcified deposit on or just under the left leaf of the diaphragm, probably an old inflammatory lesion such as a small basal empyæma, or subphrenic abscess. (The patient had a hæmatemesis many years ago, and a leakage from a gastric ulcer high up may have accounted for this.)

### PLATE XXVIII.

Fig. 1.

Fig. 2.

Fig. 3.

(2) Scar of a healed gastric ulcer half-way along the lesser curve of the stomach.

(3) Gastroptosis, and delay, the duodenal bulb lying to the left of the mid-line.

(4) Congenital mal-position and mal-development of the second and third parts of the duodenum and of the jejunum, all the coils lying to the right of the mid-line, and in the right iliac fossa.

(5) Marked coloptosis. The cæcum lay to the left of the mid-line and very deep in the pelvis, but was not fixed. There was some stasis and spasm of the circular fibres. The position of the cæcum may be partly congenital in origin, owing to abnormal mesenteric attachments.

DR. A. L. TAYLOR showed the following specimens :—

1. **Ulcerative Endocarditis of the Aortic Valve and of the Mural Endocardium,** with dilatation and hypertrophy of the left ventricle, from a syphilitic male of 47. The left ventricle is grossly dilated and somewhat hypertrophied. The cusps of the aortic valve are largely destroyed by massive friable vegetations, which have spread over the endocardium of the ventricular wall and over the ventricular aspect of the mitral valve. (Plate XXVIII., Fig. 1.)

Septic infarcts were present in the spleen and both kidneys.

It is obvious that there is gross incompetence of the aortic valve, and this has resulted in a compensatory dilatation of the ventricle in order to accommodate the regurgitant blood, as well as in hypertrophy of its muscular wall, so as to deal with a larger quantity of blood at each beat.

The aorta of this case presents also numerous raised fleshy patches (A, A), confined chiefly to its ascending portion, and particularly to the region immediately beyond the aortic ring. These patches are characteristic of *syphilitic mesaortitis.* Their great danger lies in their close proximity to the orifices of the coronary arteries, occlusion of which may in some cases result in dramatically sudden death.

2. **Ulcerative Endocarditis Affecting the Mitral Valve and Mural Endocardium,** with gross mitral incompetence, from a woman aged 31, who gave a history of chorea and heart trouble when a girl. The clinical picture was typical during the two months' treatment in hospital : loud systolic murmur, chest pains with slight hæmoptysis, renal pain with hæmaturia, attacks of severe pain in the splenic region, and purpuric rashes. At post-mortem almost every organ in the body was found to contain old or recent infarcts. (Plate XXVIII., Fig. 2.)

The mitral valve is the seat of large vegetations which have almost completely destroyed both flaps. Massive vegetations are present also in the mural endocardium of the left auricle and left ventricle, and on the papillary muscle. The aortic valve is intact.

This case contrasts with the previous one in that there is little dilatation and no hypertrophy of the left ventricle. On the other hand, the right side of the heart is enlarged, following back pressure through an incompetent mitral valve.

3. **An enormous Aortic Aneurysm** producing deep erosion of the dorsal vertebræ, from a syphilitic male aged 48. For six weeks before admission in January there was severe pain in the back and left lumbar region, stabbing and aching in character. Death occurred after ten weeks in hospital, during which time the pain became much worse and practically continuous, with progressive emaciation. X-rays showed a large fixed mass in the posterior mediastinum, which later transmitted pulsation into the epigastrium.

The specimen shows a large sacculated aneurysm arising in the posterior wall of the thoracic aorta, where its orifice is seen to be quite small, and extending backwards to form a large sac filled with laminated blood-clot. Where it has come into contact with the vertebral column this has been eroded and destroyed in a quite remarkable manner, the vertebral bodies being affected far more than the fibro-elastic intervertebral discs. (Plate XXVIII., Fig. 3.)

The ascending aorta showed the typical lesion of syphilitic mesaortitis ; the patient had chronic superficial glossitis, and a Wassermann reaction taken before death was positive.

DR. A. D. FRASER showed a series of specimens of **Carcinoma of the Stomach.**

One of these specimens was removed from a man aged 52, who died after profuse hæmatemesis. There was an ulcer on the lesser curvature near the pylorus. The outside of the stomach wall was adherent to a dense mass of omental tissue and swollen lymph glands. The base of the ulcer was formed of part of the muscular coat and the serous coat. Thickened vessels could be seen in the floor of the ulcer, and small erosions was present in their wall. There was no acute inflammatory reaction. Microscopic examination showed carcinomatous change.

## Figures and Tables

**Figure f1:**
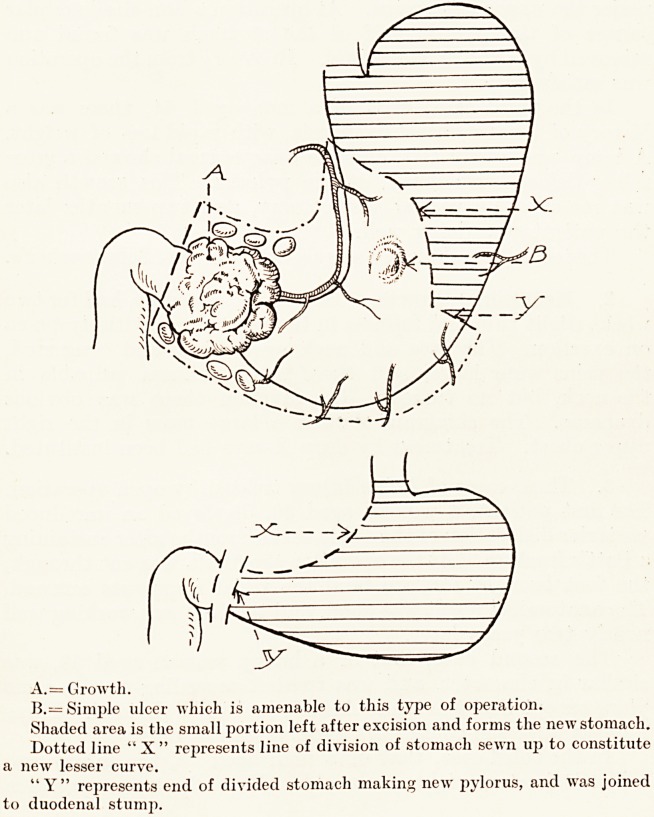


**Fig. 1. f2:**
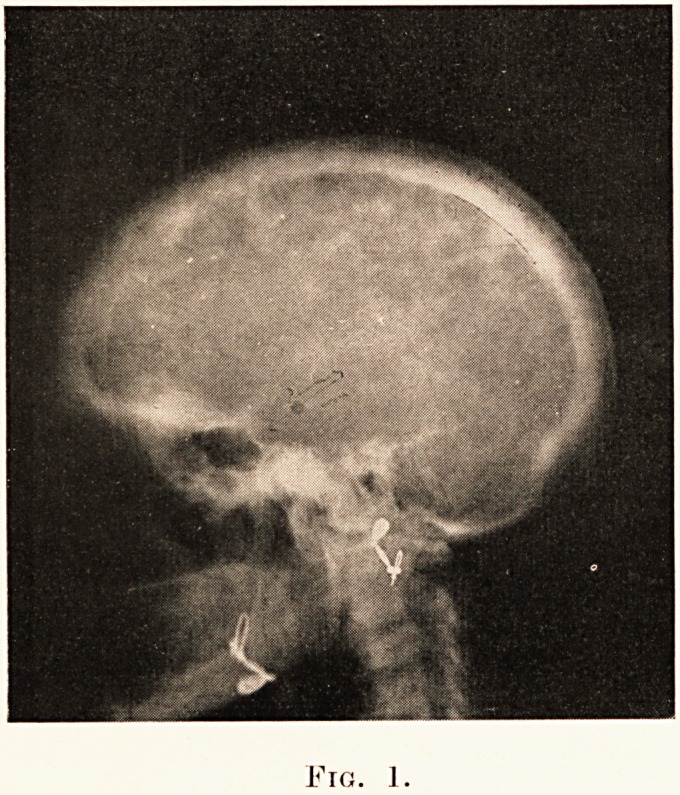


**Fig. 2. f3:**
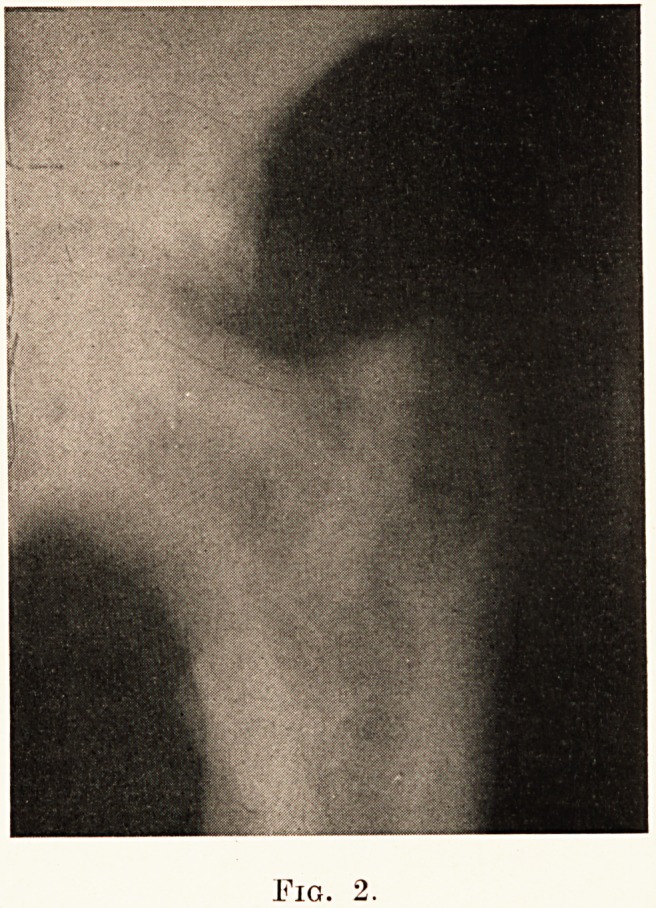


**Fig. 3. f4:**
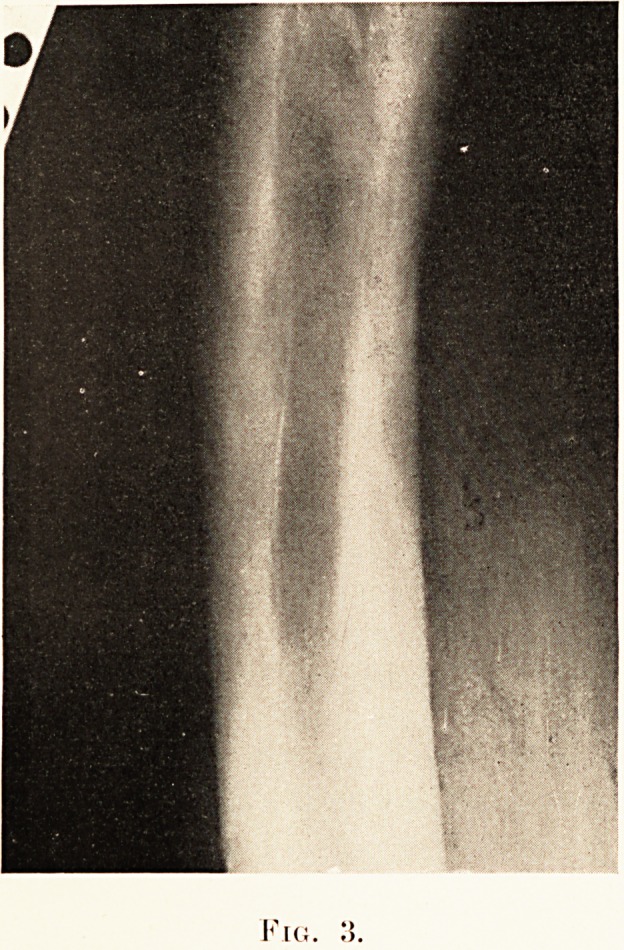


**Fig. 4. f5:**
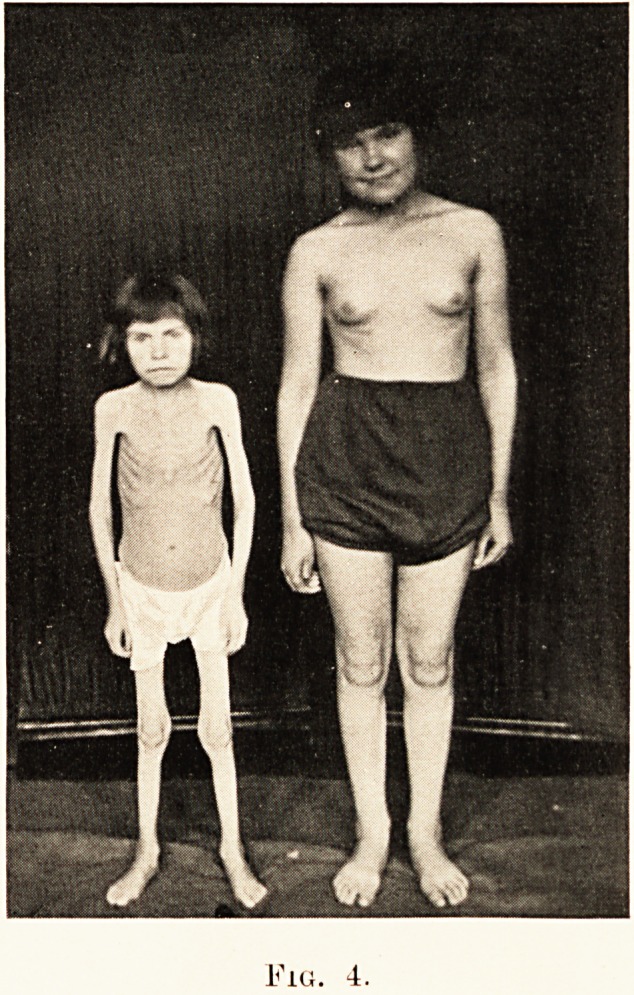


**Figure f6:**
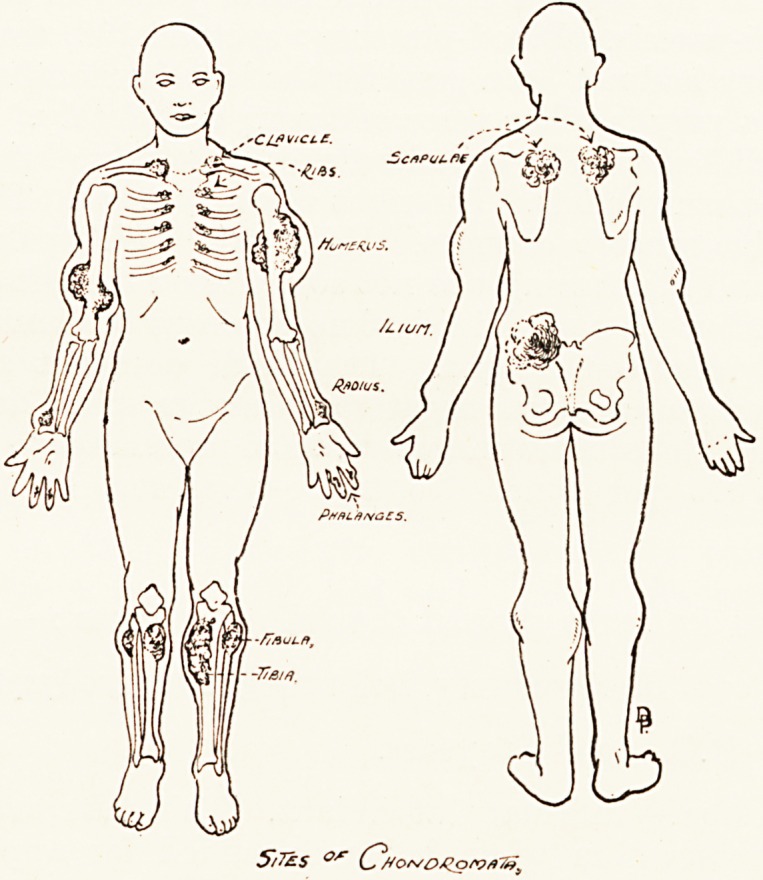


**Fig. 1. f7:**
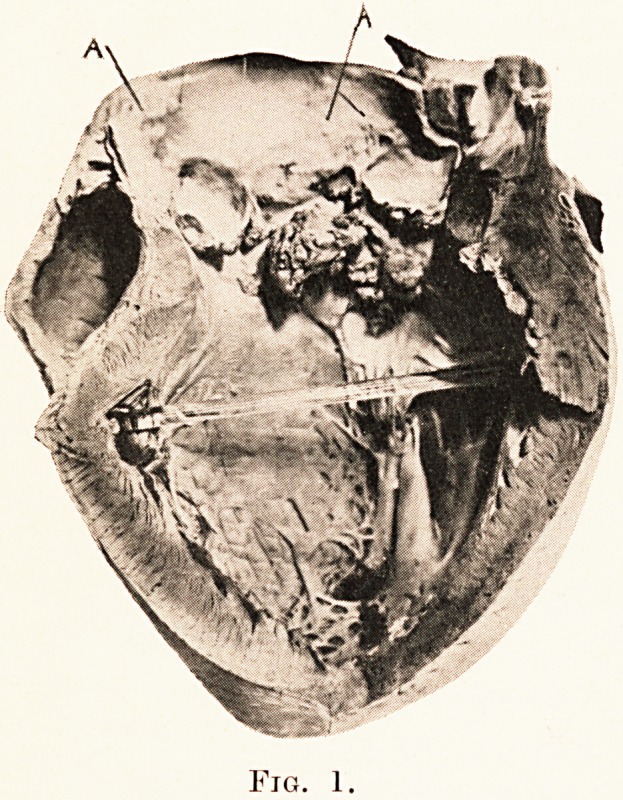


**Fig. 2. f8:**
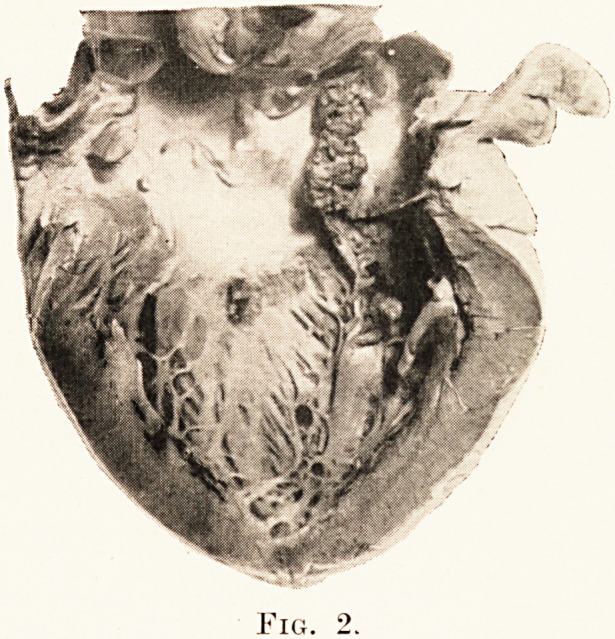


**Fig. 3. f9:**